# Fibrodysplasia ossificans progressiva with minor unilateral hallux anomaly in a sporadic case from Northern Tanzania with the common *ACVR1*c.617G>A mutation

**DOI:** 10.11604/pamj.2015.22.299.8032

**Published:** 2015-11-24

**Authors:** Mohammed Saleh, Joost Commandeur, Renata Bocciardi, Grace Kinabo, Ben Hamel

**Affiliations:** 1 Department of Paediatrics and Child Health, Kilimanjaro Christian Medical Centre, P.O. Box 2240, Moshi, Tanzania; 2 Department of Internal Medicine, Kilimanjaro Christian Medical Centre, P.O. Box 2240, Moshi, Tanzania; 3 Department of Neurosciences, Rehabilitation, Ophthalmology, Genetics, Maternal and Child Health and CEBR, Università Degli Studi di Genova, Via G. Gaslini, 5, 16147 Genova, Italy; 4 Istituto Giannina Gaslini, Medical Genetics Unit, Via G. Gaslini, 5, 16147 Genova, Italy; 5 Department of Human Genetics, Radboud university medical center, P.O. Box 9101, 6500 HB Nijmegen, The Netherlands

**Keywords:** Fibrodysplasia ossificans progressiva, heterotopic ossification, hallux valgus, recurrent ACVR1 mutation

## Abstract

Fibrodysplasia ossificans progressiva is a rare autosomal dominantly inherited disorder of connective tissue caused by mutations in the gene encoding for *ACVR1/ALK*2, a bone morphogenetic protein type I receptor. It is mainly characterized by congenital malformations of the great toes and the formation of qualitatively normal bone in extra-skeletal sites leading to severe disability and eventually death. We present a sporadic case from Northern Tanzania with a minor unilateral hallux anomaly and the common *ACVR1* c.617G>A mutation.

## Introduction

Fibrodysplasia ossificans progressiva (FOP, OMIM #135100) is a rare and disabling autosomal dominantly inherited disorder of connective tissue leading to progressive development of heterotopic ossification (HO) in extra-skeletal sites, i.e. the formation of qualitatively normal bone in skeletal muscles and other connective tissues [1]. We present a case of FOP that was diagnosed clinically, though only a minor unilateral hallux anomaly was present, in the paediatric department of Kilimanjaro Christian Medical Centre (KCMC) in Moshi, Tanzania, almost 50 years after the first FOP case was reported from Tanzania [2]. The case we present here is of interest because of the minimal and unilateral hallux abnormality in the presence of the common *ACVR1*c.617G>A mutation. Also, it is the first molecularly confirmed case of FOP in sub-Saharan Africa outside South Africa.

## Patient and observation

**Figure 1:**
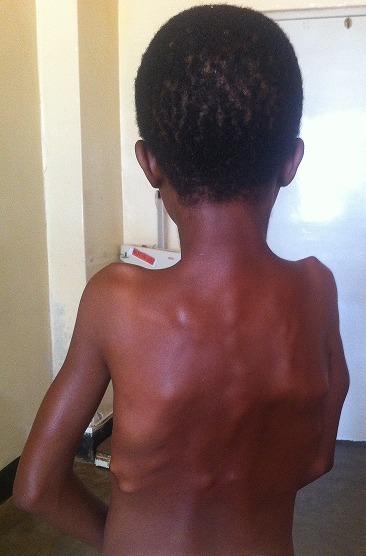
FOP lesions on the back

**Figure 2:**
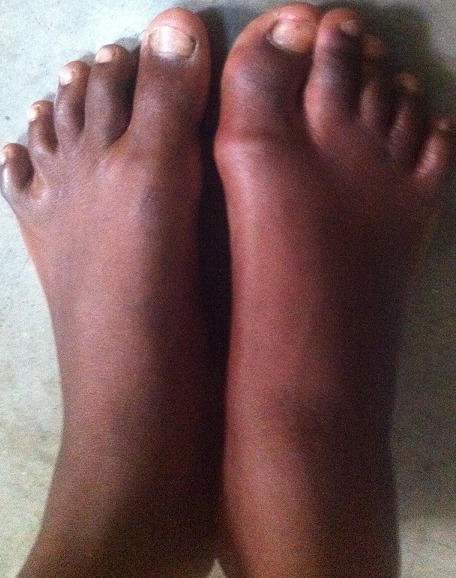
Hallux valgus of the right great toe

**Figure 3:**
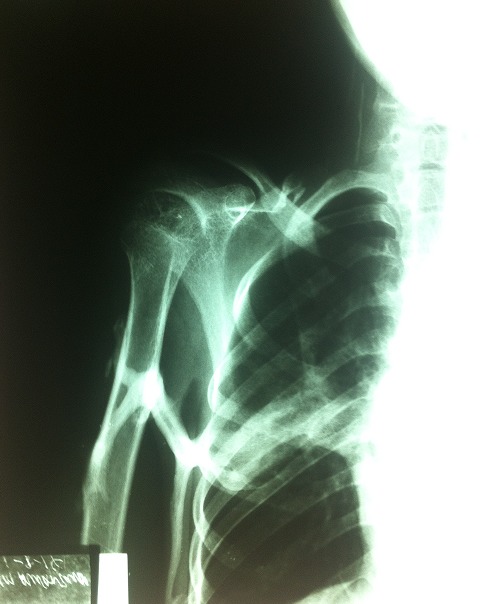
Radiograph, showing the hallux valgus of the right great toe

In October 2013, a 12-year-old girl was referred to the paediatric clinic of KCMC with fever, weakness and headache for seven days, which was diagnosed as malaria and treated accordingly. She had a history of restricted movements of arms and neck. She had pain and swelling involving the neck, shoulders and back areas, which began when she was two years of age. Over the following ten years she had repeated episodes of swelling involving the neck, shoulder and back coupled with increasing difficulty walking. At ten years of age she also started to experience difficulty with eating and talking because of decreased motility of the jaw. No history of trauma was reported. She is the seventh child in her family of eight children and the only affected in her extended family. On examination the patient was febrile with distinctive hard non-tender swellings involving the neck, shoulder and the upper part of the back (Figure 1). These abnormalities were associated with limited range of movements over the jaw, neck, shoulders and elbow joints. In addition the patient was unable to extend her legs because of contractures of the hamstring tendons. Thumbs were normal. A slight hallux valgus of the right great toe was noted (Figure 2) and confirmed radiologically (Figure 3). Additional X-rays confirmed the presence of HO lesions around the shoulder and pelvic girdles, elbows and back. A clinical diagnosis of FOP was made and a venous blood sample was obtained. Molecular analysis of the *ACVR1/ALK2*gene revealed the presence of the most frequently recurrent c.617G>A (p.Arg206His) mutation, confirming the clinical diagnosis. She was given paracetamol for the pain and continued to be seen in our clinic.

## Discussion

FOP is a rare disabling inherited disorder of connective tissue with a prevalence of 0,5 per 1 million without apparent racial, ethnic or geographical variation [1]. It is most frequently caused by a recurrent heterozygous gain-of-function mutation (c.617G>A; p.Arg206His) in the *ACVR1/ALK2* gene on chromosome 2q23, which usually occurs as a sporadic, de novo mutation, but it may be inherited from either parent [3, 4]. Other mutations may lead to a variant phenotype [5]. Mutations cause an enhanced BMP-mediated signalling leading to progressive development of HO in skeletal muscles and other connective tissues, i.e. the formation of qualitatively normal bone in extra-skeletal sites [1]. The case reported in this study appears to be sporadic, since neither parent nor other first-degree relatives are known to be affected. When present, FOP can be diagnosed clinically early in life and even prenatally [6], by the presence of short malformed halluces (monophalangism, hallux valgus and/or malformed first metatarsal). Sometimes similar malformations of the thumbs are also present. Our case had just a minor and unilateral hallux valgus and still the common *ACVR1*c.617G>A mutation. Though this has been reported before, it is rare [1]. The disease progresses with sporadic exacerbations (flare-ups) resulting in HO, which starts in the dorsal, axial, cranial and proximal regions of the body. The heart, smooth muscles and the diaphragm are most notably not affected by this disease. Flare-ups can be induced by different triggers such as trauma, surgery, diagnostic biopsies, intramuscular injections and viral infections. Also, general anaesthesia poses dangers to patients with FOP [7]. FOP is often misdiagnosed [8]. From Nigeria a case was reported where FOP was misdiagnosed as Burkitt′s lymphoma [9]. We know of another case in our hospital which was misdiagnosed as Burkitt's lymphoma and in whom a diagnostic liver biopsy contributed in our opinion to the development of HO at the abdominal wall. Another article from Nigeria demonstrated the negative effect of surgical intervention in children with undiagnosed FOP [10]. These articles show the iatrogenic harm that can be done by not recognising FOP in time. HO eventually leads to impaired mobility, weight loss due to ankylosis of the jaw and thoracic insufficiency due to costovertebral malformations. Median age of survival is 40 years [11], however, we hypothesize that this may be lower in sub-Saharan African patients due to delayed diagnosis and higher risks of trauma and infections. Currently, there is no effective prevention or cure for this disabling disease.

## Conclusion

Early diagnosis of FOP, which in cases with a minor and unilateral hallux anomaly supposes a high level of awareness, is important in order to prevent flare-ups, for example by restricting activities to reduce the risk of trauma and by reducing the number of unnecessary (invasive) investigations and interventions, thereby preventing iatrogenic harm [1, 8, 10].
